# Heed or disregard a cancer patient’s critical blogging? An experimental study of two different framing strategies

**DOI:** 10.1186/s12910-016-0115-3

**Published:** 2016-05-20

**Authors:** Niels Lynøe, Sara NattochDag, Magnus Lindskog, Niklas Juth

**Affiliations:** Stockholm Centre for Healthcare Ethics, Karolinska Institutet, 171 77 Stockholm, Sweden; Karolinska University Hospital, Stockholm, Sweden; Department of Immunology, Genetics and Pathology, Uppsala University, Uppsala, Sweden

**Keywords:** Blogging patients, Priority-setting, Decision-making, Social status, Tacit values

## Abstract

**Background:**

We have examined healthcare staff attitudes of toward a blogging cancer patient who publishes critical posts about her treatment and their possible effect on patient-staff relationships and treatment decisions.

**Methods:**

We used two versions of a questionnaire containing a vignette based on a modified real case involving a 39-year-old cancer patient who complained on her blog about how she was encountered and the treatment she received. Initially she was not offered a new, and expensive treatment, which might have influenced her perception of further encounters. In one version of the vignette, the team decides to put extra effort into both encounters and offers the expensive new cancer treatment. In the other version, the team decides to follow the clinic’s routine to the letter. Subsequently, blog postings became either positive or negative in tone. We also divided participants into value-neutral and value-influenced groups (regarding personal values) by asking how their trust in healthcare would be affected if the team’s suggestion were followed.

**Results:**

A total of 56 % (95 % CI: 51–61) of the respondents faced with a team decision to ‘do something-extra’ in encounters would act in accordance with this ambition. Concerning treatment, 32 % (95 % CI: 28–38) would follow the team’s decision to offer a new and expensive treatment. A large majority of those who received the “follow-routine” version agreed to do so in encountering [94 % (95 % CI: 91–97)]. Similar proportions were found regarding treatment [86 % (95 % CI: 82–90)].

A total of 83 % (95 % CI: 76–91) of the value-neutral participants who received the “do-something-extra” version stated that they would act as the team suggested regarding encounters, while 57 % (95 % CI: 47–67) would do so in regard to treatment. Among the value-influenced participants who received the “do-something-extra” version, 45 % (95 % CI: 38–51) stated that they would make an extra effort to accommodate the patient and her needs, while the proportion for treatment was 22 % (95 % CI: 16–27). Among those who had received the “follow-routine” version, a large majority agreed, and no difference was indicated between the value-neutral and the value-influenced participants.

**Conclusion:**

The present study indicates that healthcare staff is indeed influenced by reading a patient’s critical blog entries, largely regarding encounters, but also concerning treatment is concerned. Value-neutral healthcare personnel seem to exhibit a pragmatic attitude and be more inclined to heed and respond to a patient whose criticism may well be warranted. The study also indicates that healthcare staff is partly positive or negative to future blogging patients depending on how the issue has been framed.

For future research we suggest as a bold hypothesis that the phrase “clinical routine” might conceal power aspects masquerading as adopted ethical principles.

**Electronic supplementary material:**

The online version of this article (doi:10.1186/s12910-016-0115-3) contains supplementary material, which is available to authorized users.

## Background

In welfare states, the healthcare system is normally publicly financed and it is generally accepted that medical treatment should be allocated primarily according to medical needs, with comparable cases receiving equal treatment [1–3]. This is commonly interpreted to imply that issues like income, age, sex, ethnicity, and the ability to make oneself heard should be irrelevant. In accordance with this it has been shown that particularly the ability to make oneself heard might make healthcare staff feel frustrated [4]. Logically, then, if a patient suffering from a cancer disease is admitted and treated with greater benevolence or respect than another patient suffering from a similar condition, this would be considered unfair. Other studies have indicated that the personal values of healthcare staff might influence decision-making in a way that is contrary to official values [5, 6]. Official values are associated with principles such as beneficence, non-maleficence, justice and autonomy which are also sometimes reflected in healthcare law. A consequence of personal values influencing decision-making might entail arbitrariness in whether or not a patient receives a certain treatment [7].

In Sweden it has also been reported that healthcare staff sometimes offer VIP treatment to certain patients at the expense of others, in direct contravention of the principle that “equal cases should be treated equally” [4, 8]. Recently, a new group of notables have entered the healthcare consciousness: patients who keep blogs [9] where they describe their healthcare experiences. A cancer patient in a palliative stage might also blog about his or her experiences [10, 11]. Some of these blogs are read and commented on by thousands of followers, and are even quoted in traditional news media.

However, it has also been maintained by these so-called e-patients that a blogging patient is not only referring to his or her own case. These patients have also been described as experts on their own condition, for instance a cancer disease, and collaborate with clinicians in developing healthcare for all patients [12].

The importance of healthcare staff encounters has recently gained increased attention [13]. Healthcare staff are to display a benevolent attitude toward their patients. Moreover, respect for patients’ autonomy and shared decision-making has been stressed within the framework of patient-centred care [14, 15]. Patient-centeredness means, among other things, that the healthcare provider listens to the patient, believes what the patient says and summarizes the patient’s story. Positive outcomes have been described as a result of patient-centered care [14]. It has also been argued that encountering patients in a fair, benevolent, and respectful manner is often axiomatic to patient safety and might influence the course of a disease, its successful treatment and recovery in terms of return to work [13]. Nevertheless, it is not known to what degree encountering patients is regarded as merely an issue of etiquette, with no consequences on patients’ health and wellbeing. The question is whether or not healthcare staff actually hold encounters to be less important than the specific medical treatment.

We know that the social movement of e-patients actually influences some hospitals (such as the Mayo Clinic) within the framework of Participatory-Medicine [12]. But there are few (if any) empirical studies on whether or not a cancer patient’s critical bloggings might actually influence healthcare staff behaviour. We have chosen to conduct a vignette-based study on the issue, in an attempt to determine whether it might influence the way healthcare professionals encounter and treat their patients [16, 17]. The vignette we have chosen involves a cancer patient whose critical blog entries about encounters and treatment that was not offered attracted the attention of the team in charge of her case. Such a cancer patient might have reason to complain about both the encounters and the medical treatment. In the present case, it was indicated that the patient felt frustrated because she was initially not offered the new and expensive treatment, and that this might have influenced her perception of further encounters. The question at hand is whether staff should do something extra in response to her online criticism or simply follow routine and offer her no special attention.

The more specific objectives of the study are to examine 1) whether the participants are prepared to do something extra regarding encounters and treatment based on the criticism on a cancer patients’ blog, and whether or not staff found it right to do so in principle; 2) whether the personal values of healthcare staff (in terms of decreasing or increasing own trust if the procedure was generally accepted) influence their judgment; 3) whether different framings influence the judgment of healthcare staffs’ (the positive outcome of doing something extra or the negative outcome of following routine); and finally 4) whether nurses and physicians distinguish between encounters and medical treatment in terms of what is the most important.

## Methods and participants

In the vignette we chose it was stated that apart from palliative care, no further curative treatment was available (see Additional file 1). However, the 39-year-old cancer patient had discovered a new, expensive treatment via social media, which she requested. In order to study a possible framing effect, we used two versions of the vignette. Common to both was that the patient was described as dissatisfied how the staff had encountered her. Moreover, since the new expensive treatment was not part of the clinic’s normal routine, it was not offered. In one version, the team decided to be more accommodating when encountering the patient, as well as actually offering her the new treatment (the “do-something-extra” version). In the other, the team decided to stick to routine and hence not change how they encountered the patient or offered the new treatment (the “follow-routine” version). In English there is no exact, appropriate translation of the Swedish word *bemötande,* which was the term used in the questionnaire. In a healthcare setting, this term includes how staff express their attitudes, indicating that encounters can be perceived as more or less fair/unfair, respectful/disrespectful or benevolent/malevolent – see Table 1. “Doing something extra” might accordingly imply that healthcare staff try to be a bit more fair, respectful or benevolent. In the present case, we presuppose that the patient complained mainly because she was not offered the new and expensive treatment, and we assume that this also colored her perception of further encounters when she discovered that the new treatment option actually existed.

**Table 1:** Displays some of the core concepts used in the present paper

**A blog** is a website that contains periodically published articles and/or diary entries on a web page, with the posts usually arranged so that the most recent ones are at the top. A blog is an open diary that can be of various types, e.g. political, fashion, or disease. The blog’s visitors can typically make comments on each post, facilitating an exchange of content and information. **Encounter** in this context is related to the human interaction between healthcare staff and patients. The Swedish word for encounter (*bemötande*) implies attitudes reflected as more or less fair/unfair, respectful/disrespectful, or benevolent/malevolent. Since there is no English word that encompasses the same meaning as the Swedish one, it is important to note that when we use the word encounter it should be understood in its broader sense. **Framing effect** usually describes the effect of a *positive* or *negative* description of, e.g., clinical decision-making and perceptual judgment, often referred to as *valence* framing effects [23, 24]. It is possible to distinguish between three different framing strategies: 1) *Risky choice framing* means that different descriptions of risks result in different *outcomes* of a certain choice – the *risk preference* is affected; 2) *Attribute framing* means that different *characteristics* of an event or object influence the framing manipulation and thus the effect – the evaluation of *items* is affected; 3) *Goal framing* means that it is the *goal* of an *action* or *behavior* that is manipulated – the impact of *persuasion* is affected. **Official values** are values adopted in soft law (e.g. professional guidelines and codes of conduct) as well as healthcare law. They are often associated with principles such as autonomy, justice, beneficence and non-maleficence, as well as the principle that equal cases should be treated equally. Official values, together with factual aspects (e.g. evidence), should constitute the basis for clinical decision-making. **Personal values** are values that the individual healthcare provider might embrace but that, in a Swedish setting, should not influence clinical decision-making. Personal values might be in accordance or conflict with the official ones. Unlike the healthcare provider’s personal values, however, the patient’s values and preferences are to be considered in clinical decision-making. **Trust in healthcare** is regarded as a precondition for patients’ confidence in and reliance on healthcare providers. As a vulnerable patient, one is dependent on the healthcare provider’s goodwill (benevolence). Anything that might jeopardize one’s trust in healthcare or perception of a provider’s trustworthiness might also have an impact on the patient-provider relationship. Consequences might be considered undesirable or poor, and vice versa. Accordingly, healthcare providers might be eager to preserve patients’ trust in healthcare. If a healthcare provider perceives that an intervention does not have an impact on trust, in the present context the intervention is considered ethically neutral. **Value-influenced healthcare providers** are persons whose own trust in healthcare would be influenced (positively or negatively) if a new, expensive treatment were accepted. **Value-neutral healthcare providers** are persons whose own trust in healthcare would not be influenced if a new, expensive treatment were actually offered. Such a person usually regards the offered or requested intervention as neither good nor bad.	

In one version of the vignette, we referred to an ad hoc decision by the team to do something extra, which could then be compared with the decision to follow the clinic’s routine to the letter. We are aware that “doing something extra” might be understood as positive and thus prime the respondents’ judgment. The idea behind using these terms was to make each option possible and allow the healthcare staff to do one or the other. As a consequence of the “do-something-extra” version, the blog posts initially negative became decidedly more positive, while in the “follow-routine” version the blogger continued to express criticism or was even more critical. The differences between the two cases are accordingly related to different decisions made by the team and differences in the outcome. In this type of study the outcome is part of how it is framed and is expected to bring about framing effects.

The subsequent questions were presented as statements that were similar in both versions of the vignette, albeit adjusted to the context: 1) Like the team, I would probably have done something extra/not have done something extra when encountering the patient; 2) The team acted in the right way; 3) Like the team, I would probably have offered/not have offered the expensive new treatment; and; 4) The team acted in the right way. The response options were *agree completely* or *agree to a great extent* and *disagree completely* or *disagree to a great extent* (see Additional file 1). The reason why we used fixed response options was to be able to compare the two vignettes regarding the framing effects. “Doing something extra” was meant to illustrate a pragmatic clinical attitude, whereas the question of whether it was right to do so was intended to examine the fairness of doing it or the personal values of the participants. “Following routines” was intended to illustrate an authoritative argument in which there is little room to consider patients’ preferences in medical decision-making contrary to a pragmatic approach, which might allow a greater extent of patient participation [12].

Since the scale used was not a traditional five-point Likert scale (lacking the middle point), we conflated the responses *agree completely* and *agree to a great extent*, as well as *disagree completely* and *disagree to great extent*, respectively.

Experience from previous studies indicates that healthcare staff’s decision-making process might be value-influenced by their own personal values [5, 6, 18]. These values may even affect the staff’s factual beliefs; i.e., factual aspects may be value-impregnated. Patients and even the healthcare staff themselves, may not be aware of this. In order to study such values, we examined statements regarding how the participants’ trust in healthcare might be affected if staff chose to do something extra in response to the critical blog entries. We assumed that those whose own trust would not be influenced could be classified as a value-neutral group and those whose trust would increase or decrease as a value-influenced group regarding personal values [5, 6, 18]. In a Swedish setting, healthcare staff are not to allow their own personal values to influence their decision-making. With the study conducted in this way, the participants were not aware that we were actually studying personal values and their potential influence on how to act in such a situation. Accordingly, we also asked for the respondents’ profession, sex and age, as well as how their trust in healthcare might be affected if the patient in question were encountered and treated as described in the respective vignettes. Response options were *trust would increase*, *trust would not be influenced* or *trust would decrease*. Participants were also invited to provide comments after each answer.

On the backdrop of a pilot-study we recruited 591 nurses and 597 physicians randomly selected from a commercial company’s (Cegedim/Stockholm) list of healthcare staff purportedly employed in oncology clinics around the country. 307 nurses and 308 physicians ultimately responded. In order to study the accuracy of the company list, we asked the participants to indicate their speciality (see Table 2).

**Table 2:** The proportion (with 95 % confidence intervals) of participants who agreed completely and to a great extent that they would act in accordance with the team (upper panels) with respect to encountering the patient blogger and offering the expensive new treatment. The respondents were then asked whether it would be right to act in accordance with the team’s decision (lower panels). Proportions with 95 % confidence intervals (CI, within brackets) are provided

	I would follow the team doing something extra with respect to:	I would follow the team following routine with respect to:
*Both physicians and nurses*		
Encountering	56 % (51–61)	94 % (91–97)
Treatment	32 % (27–37)	86 % (82–90)
*Physicians*		
Encountering	51 % (43–59)	94 % (90–98)
Treatment	24 % (17–31)	90 % (85–95)
*Nurses*		
Encountering	62 % (55–71)	94 % (90–98)
Treatment	42 % (35–51)	83 % (77–89)
It would be right to:	‘Do something extra’ with respect to:	‘Follow routine’ with respect to:
*Both physicians and nurses*		
Encountering	39 % (34–44)	95 % (93–97)
Treatment	26 % (21–31)	87 % (83–91)
*Physicians*		
Encountering	34 % (27–41)	96 % (93–99)
Treatment	20 % (14–26)	92 % (87–97)
*Nurses*		
Encountering	46 % (38–54)	94 % (90–98)
Treatment	33 % (25–41)	83 % (77–89)

When distributing the two questionnaires, we randomised the participants by numbering the group of physicians and the group of nurses separately. This strategy was used in order to obtain an analogous proportion of participants from all over Sweden. Even-numbered participants received the” do-something-extra” version of the questionnaire and odd-numbered ones the “follow-routine” version. We used the Chi-2 test and presented our results as proportions with a 95 % confidence interval (CI). Non-overlapping CIs are interpreted in the present context as though a hypothesis test had been conducted; the difference would have been significant at the 0.05 level.

The participants were invited to comment after each question and provide general comments at the end of the questionnaire. The comments were analyzed using descriptive manifest content analysis [19]. The analysis was conducted inductively, without pre-set categories. First, we first read the text several times to establish a solid overall impression of its content. Next, meaning units related to the research questions were identified. We presented these meaning units and the derived condensed meaning units as contrasting, insofar as being *for* or *against* the actual behavior and it was on this basis we developed categories. For each category in favor of a course of action, we found a contrasting one against it as well as corresponding meaning units.

The study was approved by the regional research ethics committee at Karolinska Institutet (Dnr 2014/686-31/5).

## Results

The randomization process resulted in two comparable samples: a large majority of those who received the “do-something-extra” version of the vignette stated that doing so would decrease their trust in the healthcare system. A majority among those who received the “follow-routine” version claimed that their trust would not be affected, while approximately 30 % stated that their trust would increase if the patient were encountered and treated according to routine (see Table 3).

**Table 3:** The effect of the randomisation procedure in regard to response rate, sex, age and specialty/profession, comparing the two versions of the questionnaire

	Do something extra	Follow routine
Response rate	52 % (*n* = 314)	51.6 % (*n* = 311)
Profession		
Physician	53.2 % (*n* = 167)	47.4 % (*n* = 147)
Nurse	47.2 % (*n* = 147)	52.8 % (*n* = 164)
Specialty		
Oncology	86.4 % (*n* = 271)	87.6 % (*n* = 272)
Palliative care	3.6 % (*n* = 11)	2.3 % (*n* = 8)
Other	10.1 % (*n* = 31)	10.1 % (*n* = 31)
Sex		
Male	27.5 % (*n* = 88)	27.4 % (*n* = 83)
Female	72.5 % (*n* = 226)	72.6 % (*n* = 228)
Age (median, range)	50 years (25–87)	52 years (26–72)
How would this affect my own trust in health care?		
Increase	2 %	30.3 %
Not influence	28.8 %	61.2 %
Decrease	69.2 %	8.5 %
Responded		
First time	64.1 %	64.4 %
After one reminder	15.8 %	15 %
After two reminders	20.1 %	20.6 %

In the group that received the “do-something-extra” version, 56 % (95 % CI: 51–61) stated that they would make an extra effort in encountering the patient. Similarly, 39 % (95 % CI: 34–44) stated that it was right to do so – see Table 3. Offering the expensive, non-routine treatment was approved by 32 % (95 % CI: 27–37) of the same group, while 26 % (95 % CI: 21–31) found that it was the right to do (Chi-2 = 2.9, df = 1 and *p* = 0.08) (see Table 3).

As can be seen from Table 2, nurses, compared to physicians, were slightly more inclined to make an extra effort encountering the patient (Chi-2 = 3.6, df = 1 and *p* = 0.06), while they were significantly more inclined to offer non-routine treatment (Chi-2 = 11.4; df = 1, *p* = 0.0008). Compared to physicians, nurses were also more inclined to deem this right, regarding encountering the patient (Chi-2 = 4.99, df = 1 and *p* = 0.03) and treatment (Chi-2 = 6.4, df = 1 and *p* = 0.01) – see Table 2. A large majority of all participants who agreed to follow routine also asserted that it was the right thing to do. Similar proportions were identified regarding treatment, and there were only minor variations (83 and 96 %) between nurses and physicians regarding both encounters and medical treatment (see Table 2).

When classifying nurses and physicians as those whose trust would not be affected (the *value-neutral group*), we found that of those who received the “do-something-extra” version, 29 % (90/311) were classified as value-neutral while the remaining 71 % majority (221/311) were classified as *value-influenced* – see Table 1. Corresponding proportions among those who answered the “follow-routine” version 61 % (183/300) were value-neutral and 39 % (117/300) were value-influenced – see Table 1. As can be seen in Table 4, we found that significantly more (83 % [95 % CI: 76–91]) of the value-neutral participants would do something extra regarding encounters, compared to the value-influenced participants (45 % [95 % CI: 38–51]) (Chi-2 = 38.5, df = 1 and *p* < 0.001). Significantly fewer of those who were value-influenced found it right to “do something extra” (regarding encounters), whereas the group of value-neutral participants was of a similar proportion (Chi-2 = 2.79, df = 1 and *p* = 0.095) – see Table 2.

**Table 4:** Participants classified as value-neutral and value-influenced in regard to the two vignettes (*do something extra* and *follow routine*). The results are presented as proportions of those who responded in the affirmative with a 95 % confidence interval (in brackets)

	Do something extra	Follow routine
*Encounters*		
Value-influenced (*n* = 221, *n* = 117)	45 % (38–51)	90 % (84–95)
Value-neutral (*n* = 90, *n* = 183)	83 % (76–91)	97 % (95–100)
*Treatment*		
Value-influenced (*n* = 214, *n* = 112)	22 % (16–27)	88 % (83–94)
Value-neutral (*n* = 88, *n* = 172)	57 % (47–67)	85 % (80–90)
	Right to do something extra	Right to follow routine
*Encounters*		
Value-influenced (*n* = 222, *n* = 118)	25 % (19–31)	92 % (87–97)
Value-neutral (*n* = 89, *n* = 185)	73 % (64–82)	97 % (95–100)
*Treatment*		
Value-influenced (*n* = 216, *n* = 112)	17 % (12–22)	88 % (82–94)
Value-neutral (*n* = 90, *n* = 170)	47 % (37–57)	87 % (80–90)

A similar comparison regarding treatment was significant (Chi-2 = 34.8, df = 1 and *p* < 0.001) – see Table 4. Even though there was a significant difference among those who had received the “follow-routine” version regarding encounters (Chi-2 = 7.6, df = 1 and *p* = 0.006), there was no difference between the groups of value-neutral and value-influenced participants as far as treatment was concerned – see Table 4.

### Analysis of comments

A total of 1,024 comments were made - 585 in the “do-something-extra” version and 439 in the “follow-routine” version - most of which were very brief. Almost all comments were provided in relation to the specific items/questions. We identified five distinct and contrary categories – see Table 5:

**Table 5:** Categorisation of condensed comments for and against doing something extra or following routine. Some of the original comments address only encounters, others only treatment and some both encounters and treatment

For doing something extra and against following routine	For following routine and against doing something extra
*Improvement potential*	*Moral courage*
Improving encounters is always desirable, particularly if the patient’s criticism is warranted.	Improving encounters is not necessary if healthcare staff maintains a professional attitude and the moral courage to say no.
*Individualisation*	*Generalisation*
Encounters and treatment should be individualised according to the patient’s particular needs and in consideration of age and social situation (e.g. children).	All patients should be encountered and treated equally and according to medical needs.
*Allowing exceptions*	*Following rules*
It is human to make exceptions from general rules.	Follow guidelines, display moral courage and avoid reading patients’ blogs.
*Benefiting the patient*	*Harming*
Ten weeks might benefit the patient and even become more than ten weeks.	Risk of prolonging patient suffering.
*Negative consequences for staff*	*Negative consequence for the patient*
Prevent escalating negative viewpoints in the patient’s blog and avoid healthcare staff being negatively portrayed in the media.	Risk that the patient is becoming worse off/discriminated if she continues writing a negative blog.

1. “Improvement potential” versus the “moral courage” to say “no”. Among those who were prepared to do something extra, we found that the improvement potential was emphasized. They stated that we should heed the patient, since her criticism might very well be justified illustrated by the quote: *“We have to listen to critical viewpoints in order to learn”.* Those who were inclined to follow routine seemed to be rather sure that there was no room for improvement and simply maintained that it was: *“sad to be exposed in media, but we have to tolerate it.”*
2. “Individualization” versus “generalisation”. Those who were inclined to do something extra were also prepared to see the needs of the individual patient, which can be illustrated by the subsequent statement: *“This patient apparently had special needs, which should be the point of departure”.* Those who followed routine tended to use general statements such as: *“What would happen if all the patients blogged - do something extra for everybody?”*
3. “Allowing exceptions” versus “following the rules”. The group who would be prepared to do something extra were inclined to make exceptions and might state, for instance, that: *“We could offer treatment even though it’s not part of the routine”;* whereas those following routine stated that: *“Equal cases should be treated equally regardless of sex, age, etc.”*;
4. “Benefit” versus “harm”. Those who were inclined to do something extra were also inclined to stress the chance that the patient’s survival time might be prolonged more than 10 weeks, illustrated by the following quote: *“Benefitting the patient should take precedence – a blog post shouldn’t have influence*”. On the other hand those who would follow routines stressed the opposite implying that prolonging life also might prolong suffering. This can be illustrated by the following quote: *“It depends on how it would benefit the patient – the suffering could also be prolonged*.”
5. “Negative consequences for staff” versus “negative consequences for the patient”. Those who would do something extra were afraid of the personal effects of continued negative blog posts, illustrated by the quote: *“(I) would feel uncomfortable which might result in more impersonal encounters”*. Those who would follow routine were afraid of the consequences for the patient: *“The patient shouldn’t be punished because of the blog post.*”

## Discussion

### Can a patient’s critical blog post actually affect decision-making?

The most conspicuous result of the present study is the fact that participants who received the “do-something-extra” version were prepared to actually do something extra in reaction to reading the critical blog entry, both in the manner in which they may previously have encountered the patient and by offering the new, expensive treatment. A somewhat smaller proportion also felt it was right to do so. Considering it *right* to do something extra in this context might indicate that the participants found that the act was fair and in accordance with the beneficence principle or/and the autonomy principle, which is also illustrated in their comments.

As shown in Table 2, a large majority of those who would do something extra claimed that their trust in healthcare would decrease if doing something extra became practice, while only few stated that their trust would increase. If we assume that the former implies that doing something extra is a bad thing, we might conclude that even though it is bad, it might be the lesser of two evils. In other words, the participants might have considered doing something extra as an exception to the principle that equal cases be treated equally. If we assume that this kind of critical patient blogger is relatively rare, making the odd exception will probably not have any lasting impact on the healthcare system, even in cases when there are no substantial grounds for the patient’s criticism. But if we assume that the inclination to do something extra might be regarded as a pragmatic attitude, it might also indicate that this minority of pragmatic healthcare staff were more open to letting patients’ preferences count quite in accordance with the new social movement of “participatory based medicine” [12]. According to this movement of patients (so-called e-patients), being active on social media does not mean speaking solely for oneself – they are actually speaking for all patients in a similar position [12]. These e-patients are likely not a tiny group – it seems to have become a movement with an increasing number of followers, and with increasing importance. At the Mayo Clinic, Dave deBronkart, a former e-patient was appointed as Visiting Professor in Internal Medicine in 2015 [12]. Furthermore if we assume that the care of the present patient was actually suboptimal, it is not a question of making an exception but rather a reason to strive to improve the care – in other words, it could be considered a wake-up call and highlight the importance of the “improvement potentiality” as illustrated in the qualitative content analysis (Table 5).

Among those who received the “follow-routine” version, a large majority deferred to the rules. According to their comments, the main argument for following routine is that all patients should be received and treated equally, and the treatment should not depend on how loudly a patient can yell. Their comments also indicated that they feared that deviating from routine might result in preferential treatment and increase the risk of harm.

The recipients of the “follow-routine” version did not distinguish between following the team’s decision and finding this action to be right. As such, their reasoning seems more consistent than that of the respondents to the “do-something-extra” version, who clearly distinguished between what they would do and what they considered to be the right course of action. However, albeit consistent, the former group’s reasoning may disguise a moral problem. It is well known that demanding patients are sometimes considered “difficult”. As a result, healthcare personnel might react by stating that it is the *staff* who should determine what to do and how to do it, not the *patient* [12]. This is quite contrary to the participatory-based medical movement, represented by e.g., Dave deBronkart [12]. Perhaps “routine” serves as barrier against the imprecations of patients, even when they are legitimate or pinpoint a problematic healthcare issue. In other words, staff may be hiding what they consider a legitimate claim to power and decision-making behind a mask of “clinical routine”. Reference to “clinical routine” might well conceal morally dubious standpoints and this deserves further investigation.

A more benevolent interpretation of referring to routine might be that healthcare staff will evaluate a patient’s request for life-prolonging treatment at the end of life. As stated by Winkler et al., it is not a question of either futility or respecting an extreme version of patient-autonomy [20]. Referring to routine might well represent a systematic review of the effect of the new treatment, its potential side-effects (risk of harm), and whether or not the patient understands his or her medical situation, and is a realistic mean for understanding the benefit-harm ratio and, finally, considering the cost. Such a systematic review might diminish the risks of being influenced by the healthcare staff’s personal values.

### Do the personal values of healthcare staff affect clinical judgment?

In the present study the value-neutral group consisted of those who were prepared to go beyond official values and actually offer the patient something extra, while the value-influenced group tended to make decisions in accordance with official principles and established routine. Compared to the group whose personal values influenced their judgment, a majority of the value-neutral group also found it right to do something extra regarding both encounters and medical treatment. A minority of the value-influenced group were prepared to do something extra, while significantly fewer found it right to so.

One explanation for this might be that the group of value-neutral respondents have provided a more pragmatic attitude toward patients; i.e. they are prepared to help patients regardless of whether they are “difficult or demanding” [5, 6]. These findings are consistent with another study, in which value-neutral physicians were also prepared to help a young female who requested hymen restoration due to honor-related threats [5]. Correspondingly, those who were classified as value-influenced tended to refuse her request under any circumstances.

These results are also reflected in the qualitative results. Since the majority of the value-neutral group both found it right and were prepared to do something extra they also stressed the improvement potential, allowing exceptions and benefitting the individual patient – all aspects in accordance with patient-centered care, shared decision-making, as well as patient-empowerment considering patients’ preferences [12, 21]. Those who were inclined to follow routine were of the opposite opinion and stressed their own moral courage, following rules, and the non-harming aspects.

However, the present results do not explain why the number of ethically neutral respondents was twice as great than among those who received the “follow-routine” vignette compared to those who received the “do-something-extra” version. But if we presuppose that the more controversial an issue is the fewer the number of value-neutral participants and vice-versa, we might also assume that “doing something extra” for a blogging patient is more controversial than simply following routine. This might in turn explain the different distribution of value-neutral participants between the different studies [22]. To sum up, the study indicates that personal values influence decision-making regarding a blogging patient’s request, although they are disguised as public values and routine.

### What is the effect of framing differently?

The two versions of the vignette resulted in different framing effects. In one vignette, the team made an ad hoc decision to change the way they encountered the patient and offered the new, expensive treatment. As a result, the outcome changed (the blogging became more positive). In the other vignette, the team decided to follow routine and the outcome did not change (continued to be negative). Comparing the two vignettes referring to the team’s decision in both cases may result in no difference in framing effects in this respect. But although the initial negative blog entries were common in both vignettes, it is not quite clear what type of framing effect we are dealing with [23, 24]. The outcome in terms of discontinued negative blog entries seems to have influenced participants’ response-pattern somewhat due to the positive description of “doing something extra”. If we assume that the goal was to discontinue the patient’s negative blog posts, we might classify the effect on the participants’ choice as goal-framing manipulation. But the outcome, in terms of continued negative blogging, does not seem to have influenced the participants’ response in “following clinical routine”. The question is whether or not “following clinical routine” is something positive, and perhaps even more positive compared to “doing something extra” when this “something extra” goes beyond clinical routine. It is clear from the results that these two framings actually differ, although they might both be referred to as attribute framings. We suggest that the two vignettes seem to contain all three framing manipulations as suggested by Levin, Schneider and Gaeth [23].

A complementary *prima facie* explanation could be that since the clinical team decided to proceed in accordance with routine, the procedure is thus considered the normal way to proceed, and all other alternatives are considered extraordinary and possibly unsafe or perhaps even harmful. This point of view is also stressed in the qualitative analysis of those who defended “following routine” and being against “doing something extra”.

We suggest that the term “routine” might include procedures, power and value aspects – including both official and personal values, that deserve to be studied further.

### Do nurses and physicians distinguish between encounters and treatment?

Even though there were only some minor differences between physicians and nurses regarding “doing something extra”, it is interesting that compared to physicians, nurses were significantly more inclined to offer treatment. This finding might reflect the fact that the final decision of whether or not to offer treatment is in the hands of the physician. However, it might also reflect the fact that within a clinical team we might find different opinions, attitudes and focuses, which might cause tension in the clinical teamwork [25].

Both nurses and physicians made significantly different choices between doing something extra when dealing with patients, on the one hand, and doing the same regarding treatment on the other. When treating a cancer patient, it appears that ensuring that the patient is receiving effective and safe treatment is of utmost importance in the minds of the staff, while encountering patients is perceived as merely a matter of being polite and not directly rude; in other words, encounters might be understood as simply an issue of professional etiquette [13]. Studies indicate, however, that cancer patients find that respectful encounters can serve to accelerate recovery their rates in terms of facilitating return to work [13].

### Suboptimal encounters and treatment?

In the vignette, whether or not the patient had actually received optimal care was not explicitly stated. The only statement presented, common to both versions of the vignette, was the fact that the patient was initially not informed about or offered the new expensive treatment. We assumed that this might have influenced the patient to perceive the encounters as negative, at least when she discovered that the treatment option existed. But if we assume that the care provided was indeed optimal, the patient’s criticism might be considered unreasonable. Several participants commented that they always strive to do something extra and that there was no room for improvement. But if we assume that the patient’s criticism was justified and indicated that her care had been suboptimal, this reasoning must be changed. Rather than regarding going the extra mile for this patient as preferential treatment, it could be seen as doing what should have been done in the first place. Several participants from both groups used the phrase “learn something” or referred to “a wake-up call” when commenting on the patient’s negative blogging. These participants might have assumed that there was room for potential improvement, at least when encountering the patient, and considered it only right that the team make an extra effort.

If we accept that the patient’s encounters with healthcare staff were indeed suboptimal, this is yet another argument in favor of heeding and accommodating her opinion. If a patient in a Swedish hospital is dissatisfied with the care received, he or she can register a complaint with a variety of authorities. But processing these complaints might take months, if not a year or longer, thus, this is no real option for a cancer patient at the end of life. Blogging might be considered a last resort or, at least, a more direct and efficient way to be heard and optimize the care and treatment received. The question remains as to how the healthcare system can preclude patient dissatisfaction in the first place. The new of participatory-medicine-movement indicates that there is a need for more systematic review of e-patients’ blogs as well as complaints from the traditional authorities [12].

When there is no further curative treatment to offer a patient and palliative care is the only remaining option, he or she can easily become despondent. Some might even react with anger toward healthcare staff. Numerous participants commented on this issue, and suggested that staff should spend time listening to the patient more carefully and provide the information about the diagnosis and prognosis in an individualized manner. Several participants suggested that this patient might have special requirements in the form of psychological support and individualized information. If she is actually in crisis, her blog may be a desperate cry for help that must not be disregarded. The suggestion by Winkler et al., for a more systematic approach when dealing with cancer patients who demand new (not yet proven) treatment might also facilitate and help both clinicians and patients [20].

### Strengths and weaknesses

This kind of experimental study has been repeated in another setting with similar framing effects and its results correspond with those of a previous study conducted in a Swedish setting [5]. Whether it is reasonable to choose a vignette referring to a new, expensive treatment that, while available, has not yet become accepted routine and has a limited life-prolonging effect is a matter for debate [20]. Recently, drugs for the treatment of melanoma and advanced prostate cancer were approved by regulatory agencies, but their actual clinical availability was delayed and treatment withheld due to their high cost, despite the unequivocal survival benefits observed in blinded randomized trials [26].

While it is considered a promising tool for assessing professional practice variation [17, 27], it may prove difficult to generalize conclusions from vignette-based studies. The present study should be understood as a first step into the unexplored terrain of patient blogging and its impact on healthcare staff.

We are aware that the two vignettes were different regarding framing, but even though the questionnaires were tested on a pilot- basis we were not able to predict the size of the difference. While the ambition was not to provide too many types of framings in the vignettes, we must admit that we were not aware that following routines as the team had decided had such an impact on healthcare providers. This is a weakness of the study. But as a spin-off effect, we suggest as a hypothesis that the term “routine” conceals several aspects not yet studied. We believe that this could be a fruitful point of departure for future research.

Although the response rate of 52 % was low compared to other questionnaire-based studies distributed among Swedish healthcare personnel, it was at roughly the same level as in another study of similar design [28]. Moreover, a recent study exploring Swedish oncologists’ psychosocial attitudes, beliefs and perceptions yielded a similar response rate [29].

Although the incidence of patient bloggers like the one described in the present vignette is likely quite low, when they do in fact occur they might give rise to discussion and controversy within the team or at the clinic. We believe the present study can assist concerned teams in analyzing and discussing the issue.

## Conclusion

The present study indicates that healthcare staff are influenced by patient bloggers’ criticism. Value-neutral healthcare personnel seem more pragmatic and more inclined to listen to and help a patient whose complaints might be warranted. Although the incidence of critical bloggers might be rare, it is important to consider patients’ participation and preferences within the framework of participatory-medicine. We suggest that the wording “clinical routine” might conceal power aspects masquerading as acceptable ethical principles, and that the concept and its use should be more closely investigated.

## Additional file

Additional file 1: The two applied questionnaires. (DOC 37 kb)
