# Supplementary material for: Heed or disregard a cancer patient’s critical blogging? An experimental study of two different framing strategies
Source: BMC Med Ethics. 2016 May 20;17:30. doi: 10.1186/s12910-016-0115-3 (PMC4874013; doi:10.1186/s12910-016-0115-3)
Supplement: Additional file 1: — The two applied questionnaires. (DOC 37 kb) [file 12910_2016_115_MOESM1_ESM.doc]

A 39-year-old woman recently passed away after battling breast cancer. The patient had been diagnosed five years earlier. At an early stage in her treatment, she began blogging about her disease and experience of healthcare in social media. The patient wrote about how she was encountered by healthcare staff and the treatment she was offered. The posts were sometimes fairly critical; for example, she named members of the staff that she believed encountered her in a negative way or would not provide the treatment she requested.

The blog came to be read by thousands of people and was even quoted in broadcast media. The treatment team recognized the patient’s posts and discussed the best way of accommodating her. Its members agreed to make an extra effort into their encounters and treatment and to do their best to satisfy the patient’s demands. The team also agreed to offer an expensive new treatment that could possibly prolong life for up to ten weeks – a treatment that though available was not yet part of clinical routine.

The treatment team’s new approach was reflected in the blog, containing multiple positive portrayals of the staff involved and the healthcare system in general.

*Based on the background information given above, check the box that best matches your opinion:*

1) Like the staff, I would most likely have made an extra effort in my encounters with the patient.

☐ Agree completely ☐ Agree to a great extent ☐ Disagree to a great extent ☐ Disagree completely

Comments:

2) The staff did the right thing by making an extra effort when encountering the patient.

☐ Agree completely ☐ Agree to a great extent ☐ Disagree to a great extent ☐ Disagree completely

Comments:

3) Like the staff, I would most likely have offered the patient the treatment that is not part of the routine.

☐ Agree completely ☐ Agree to a great extent ☐ Disagree to a great extent ☐ Disagree completely

Comments:

4) The staff did the right thing in offering treatment that is not part of the routine

☐ Agree completely ☐ Agree to a great extent ☐ Disagree to a great extent ☐ Disagree completely

Comments:

5) My trust in healthcare would most likely be affected if patient bloggers were encountered and treated as in the example above.

☐ My trust would increase ☐ My trust would not be affected ☐ My trust would decrease

6) I believe that the general public’s trust in healthcare would be affected if patient bloggers were encountered and treated as in the example above

The general public´s trust would ☐ increase ☐ not be affected ☐ decrease

Finally, a few questions about yourself:

My profession is: Physician/Nurse

My main area/specialty is:

Age: ____

Sex: Male/Female

Comments:

**Thank you for taking part!**

A 39 years old woman recently died in breast cancer. The patient had got the diagnosis five years earlier. Early on she started to write about her disease and experiences of health care in social media on her blog. The patient wrote about how she was encountered and the treatment she was offered. The posts were sometimes fairly critical: she did for example name the staff that she believed encountered her in a negative way or that did not give her the treatment she requested.

The blog came to be read by thousands of people and were at times quoted in broadcast media. The treatment team recognized the patient’s posts and discussed the best way of accommodating her. Its members agreed upon not to do let the patient’s blog influence encounters and treatment. The team decided among other things to deny the patient´s request for expensive new treatment that could prolong her life for up to ten additional weeks – an available treatment, but not yet part of the routines.

The treatment team’s approach was reflected in the blog containing multiple negative portrayals of the staff and the healthcare system in general.

*Based on the background information given above, check the box that best matches your opinion:*

1) Like the staff, I would most likely not have changed the encountering of the patient

☐ Agree completely, ☐ Agree to a great extent, ☐ Disagree to a great extent, ☐ Disagree completely

Comments:

2) The staff did the right thing when not changing the encountering of the patient

☐ Agree completely, ☐ Agree to a great extent, ☐ Disagree to a great extent, ☐ Disagree completely

Comments:

3) Like the staff, I would most likely not have offered the patient the treatment that is not part of the routine

☐ Agree completely, ☐ Agree to a great extent, ☐ Disagree to a great extent, ☐ Disagree completely

Comments:

4) The staff did the right thing when they did not offer the treatment that is not part of the routine

☐ Agree completely, ☐ Agree to a great extent, ☐ Disagree to a great extent, ☐ Disagree completely

Comments:

5) My trust in healthcare would most likely be influenced if patient bloggers were encountered and treated as in the example above

☐ My trust would increase, ☐ My trust would not be affected, ☐ My trust would decrease

6) I believe that the general public’s trust in health care would be influenced if blogging patients were encountered and treated like in the example above

The general public´s trust would ☐ increase, ☐ not be affected, ☐ decrease

Finally, a few questions about yourself:

My profession is: Physician/Nurse

My main area/specialty is:

Age: ____

Sex: Man/Female

Comments:

**Thank you for participating!**
